# Gga-miR-219b targeting BCL11B suppresses proliferation, migration and invasion of Marek’s disease tumor cell MSB1

**DOI:** 10.1038/s41598-017-04434-w

**Published:** 2017-06-26

**Authors:** Chunfang Zhao, Xin Li, Bo Han, Zhen You, Lujiang Qu, Changjun Liu, Jiuzhou Song, Ling Lian, Ning Yang

**Affiliations:** 1 Department of Animal Genetics and Breeding, College of Animal Science and Technology, China Agricultural University, Beijing, 100193 China; 2 College of Animal Science and Veterinary Medicine, Tianjin Agricultural University, Tianjin, 300384 China; 3 Division of Avian Infectious Diseases, Harbin Veterinary Research Institute of Chinese Academy of Agricultural Sciences, Harbin, 150001 China; 4 Department of Animal & Avian Sciences, University of Maryland, College Park, Maryland, 20742 United States

## Abstract

Marek’s disease (MD), caused by Marek’s disease virus (MDV), is a lymphotropic neoplastic disease. Previous miRNAome analysis showed gga-miR-219b was significantly downregulated in MDV-induced lymphoma, and one of its potential target genes, B-cell chronic lymphocytic /lymphoma 11B (BCL11B) was predicted. In this study, we further investigated the function of gga-miR-219b, and the gain/loss of function assay showed gga-miR-219b inhibited cell migration and reduced cell proliferation by promoting apoptosis not by cell cycle arrest. Gga-miR-219b also suppressed expression of two cell invasion-related genes MMP2 and MMP9. The results indicated suppressive effect of gga-miR-219b on MD tumorigenesis. The gene BCL11B was verified as a direct target gene of gga-miR-219b. RNA interference was performed to block BCL11B. As expected, the effects triggered by BCL11B downregulation were in accordance with that triggered by gga-miR-219b overexpression, suggesting that BCL11B was a stimulative regulator of MD transformation. Moreover, both gga-miR-219b and BCL11B influenced the expression of Meq gene, the most important oncogene in MDV. Additionally, gene expression level of anti-apoptotic genes BCL2 and BCL2L1 was downregulated and pro-apoptotic gene TNFSF10 was upregulated in MSB1 cells with gga-miR-219b overexpression or BCL11B knockdown, which suggested gga-miR-219b promoted cell apoptosis via regulating gene expression in the apoptosis pathways.

## Introduction

Marek’s disease is a critical disease in poultry, characterized by immunosuppression, neurological disorders and rapid-onset CD4^+^ T-cell lymphoma^1^. It is also a good biomedical research model for virus-induced lymphoma disease^2, 3^. Recently, numerous researchers have reported that many microRNAs, including miR-181a, miR-26a, and miR-219b, are implicated in virus-induced tumors and play important roles^4–6^.

MicroRNA (miRNA) is small non-coding single-stranded RNA (approximately 22 nucleotides) that play important roles in regulating various biological processes, including cell proliferation, differentiation, development, apoptosis and tumorigenesis^7^. MiRNAs regulate expression of target protein-coding genes at the post-transcriptional level by interacting with the 3′-untranslated region (UTR) of mRNA or affecting translation of mRNA^8^. Currently, an increasing number of studies are investigating the involvement of miRNAs in MD. Both host and viral miRNAs related to MD tumorigenesis have been broadly reported. MiR-150 and miR-223 were downregulated in MDV-transformed cell lines, whereas downregulation of miR-155 was specific for MDV-transformed tumor cells^9^. Li *et al*. (2014) showed significant differential expression of 79 miRNAs that were regarded as sensitive to infection by MDV and found two clades of chicken miRNAs that were considered to be signatures for MDV infection and tumorigenesis^10^. Gga-miR-26a was downregulated in MDV-induced tumors^11^ and mediated IL-2 expression in transformed avian lymphocyte lines^5^. In addition, gga-miR-26a also targeted never in mitosis gene A-related kinase 6 (NEK6) and suppressed MD lymphoma cell proliferation^12^. Gga-miR-15b was reduced in MDV-infected susceptible 7_2_ chickens and splenic tumors and modulated the expression of activating transcription factor 2 (ATF2), which might be related to MD resistance/susceptibility^13^. Gga-miR-181a targets v-myb myeloblastosis viral oncogene homolog-like 1 (MYBL1) and inhibits proliferation of MSB1 cells^14^. Gga-miR-130a targets homeobox A3 (HOXA3) and MyoD family inhibitor domain containing (MDFIC), and gga-miR-103-3p targets cyclin E1 (CCNE1) and transcription factor Dp-2 (TFDP2). Both gga-miR-130a and gga-miR-103-3p inhibit proliferation of MSB1 cells^15, 16^. For viral miRNAs, Burnside *et al*. (2006)^17^ found eight miRNAs encoded by MDV. Five of them flanked the Meq oncogene, and therein, three were mapped to the latency-associated transcript (LAT) region of the genome in MDV-infected CEF cells^17^. Mdv1-miR-M4-5p, a functional orthologue of gga-miR-155, is critical for the oncogenicity of MDV^18^, and it activates the oncogene c-Myc by targeting TGF-β binding protein 1 (LTBP1) and suppressing the TGF-β signalling pathway^19^.

Our previous study investigated miRNA expression profiling between MD lymphoma and non-infected samples of chickens using Solexa deep sequencing, which unveiled that gga-miR-219b was downregulated in MD lymphoma samples compared with its expression in non-infected ones^20^. In this study, we verified that BCL11B was a target gene of gga-miR-219b and preliminarily investigated the function of gga-miR-219b and BCL11B in Marek’s disease tumor cell line.

## Results

### Gga-miR-219b inhibited MSB1 cell proliferation by promoting apoptosis, not by cell cycle arrest

**Figure 1:**
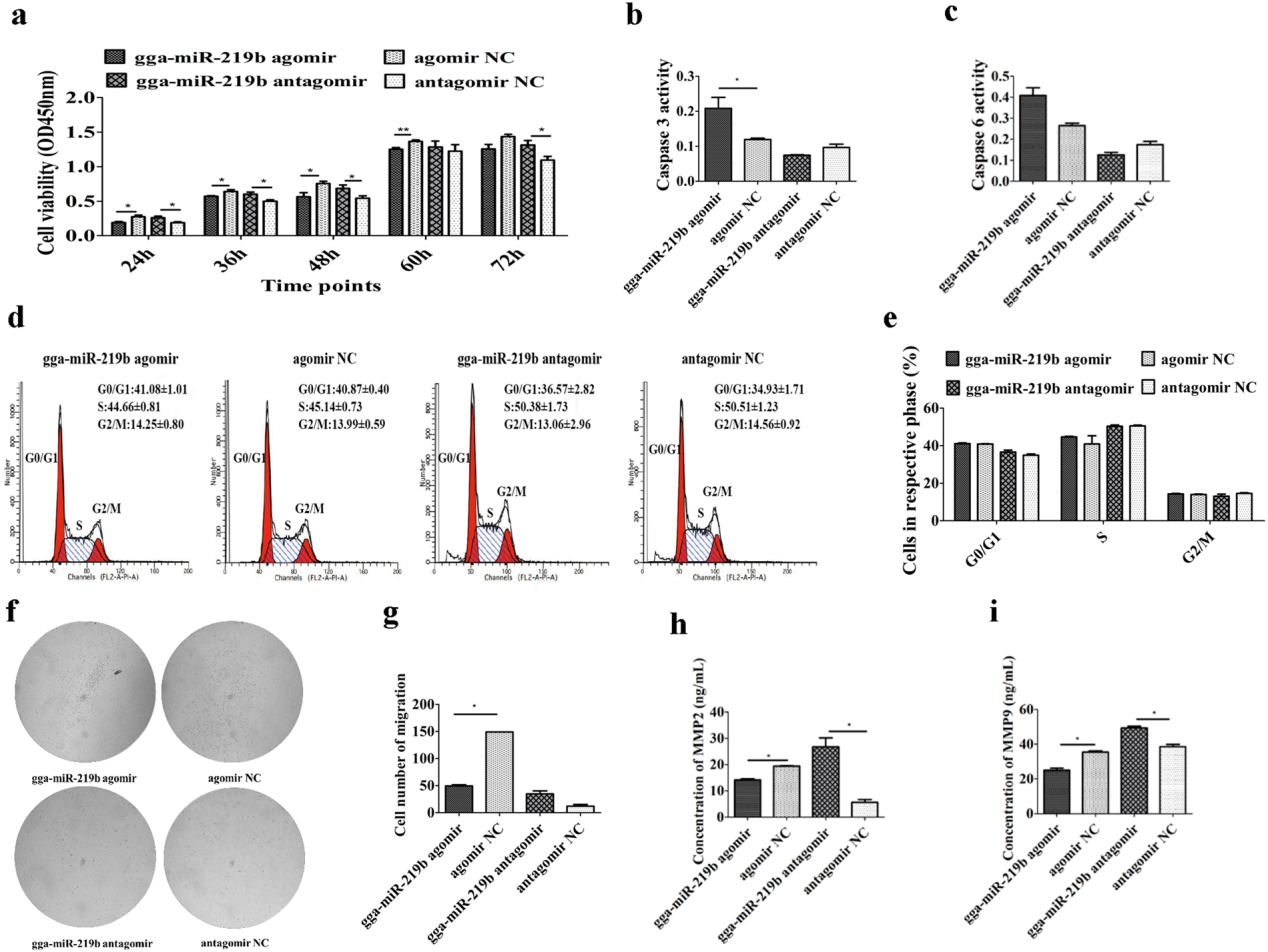
Effect of gga-miR-219b on cell proliferation, migration and invasion in MSB1 cells. (**a**) Effect of gga-miR-219b on MSB1 cell proliferation. Cell proliferation was detected by a CCK-8 assay at 24 h, 36 h, 48 h, 60 h and 72 h after transfection with the gga-miR-219b agomir, antagomir or respective NC (n = 5). (**b,c**) Effect of gga-miR-219b on MSB1 cell apoptosis. The activity of caspase-3 (**b**) and caspase-6 (**c**) was detected after transfection with the gga-miR-219b agomir, antagomir or respective NC (n = 3). (**d**) Representative histograms depicting cell cycle profiles of MSB1 cells transiently transfected with the gga-miR-219b agomir, antagomir or respective NC (n = 3). (**e**) Proportion of cells in various phases of the cell cycle (n = 3). (**f**) Representative images depicting cell migration profiles of MSB1 cells transiently transfected with the gga-miR-219b agomir, antagomir or respective NC (n = 2). (**g**) Effect of gga-miR-219b on MSB1 cell migration. Transwell migration assay performed after transduction of the gga-miR-219b agomir, antagomir or respective NC (n = 2). (**h,i**) Protein level of MMP2 (**h)** and MMP9 (**i)** after transduction of the gga-miR-219b agomir, antagomir or respective NC (n = 3). Differences between the two groups were analysed by Student’s *t*-test with the SAS system. The data are expressed as the mean ± S.E. *P < 0.05. **P < 0.01.

The viral load of MDV was 3 × 10^^8^ ± 5 × 10^^6^ copies/100 ng in MSB1 cells RNA determined by quantitative real time PCR (qRT-PCR). The MSB1 cells were transfected with the gga-miR-219b agomir or antagomir to simulate overexpression or inhibition of gga-miR-219b, respectively. The results showed that cell proliferation was lower in cultures at 24 h, 36 h, 48 h and 60 h post-agomir transfection than in the negative control (NC) transfection group. In contrast, cell proliferation was remarkably higher at 24 h, 36 h, 48 h and 72 h after antagomir transfection than in the NC transfection group (Fig. 1a). Overexpression of gga-miR-219b tended to promote apoptosis, while inhibition of gga-miR-219b markedly reduced apoptosis at 48 h post-transfection (Supplementary Fig. S1). The activity of downstream effectors caspase-3 and caspase-6 was increased in the agomir transfection group, while their activity was decreased in the antagomir transfection group at 48 h (Fig. 1b,c). Furthermore, regardless of gga-miR-219b agomir or antagomir transfection, it had no effect on the cell cycle at 48 h post-transfection (Fig. 1d,e).

### Gga-miR-219b inhibited MSB1 cell migration and invasion

The migration cell number was significantly decreased when MSB1 cells were transfected with agomir, while there was an upward trend in cell migration when cells were transfected with antagomir (Fig. 1f,g). The expression levels of two genes, MMP2 and MMP9, that are closely related to cell invasion were examined by qRT-PCR, ELISA and western blotting to evaluate the effect of gga-miR-219b on cell invasion. mRNA expression of MMP2 was significantly lower at 24 h, 48 h and 72 h post-agomir transfection than in the NC transfection group. When gga-miR-219b was inhibited by antagomir, MMP2 expression was upregulated at 48 h and 72 h. The expression of MMP9 was markedly reduced in the agomir transfection group at 24 h (Supplementary Fig. S2). MMP2 and MMP9 protein levels were significantly decreased post-agomir transfection, while their levels were significantly increased post-antagomir transfection at 48 h (Fig. 1h,i, Supplementary Fig. S14).

### BCL11B was a target gene of gga-miR-219b

**Figure 2:**
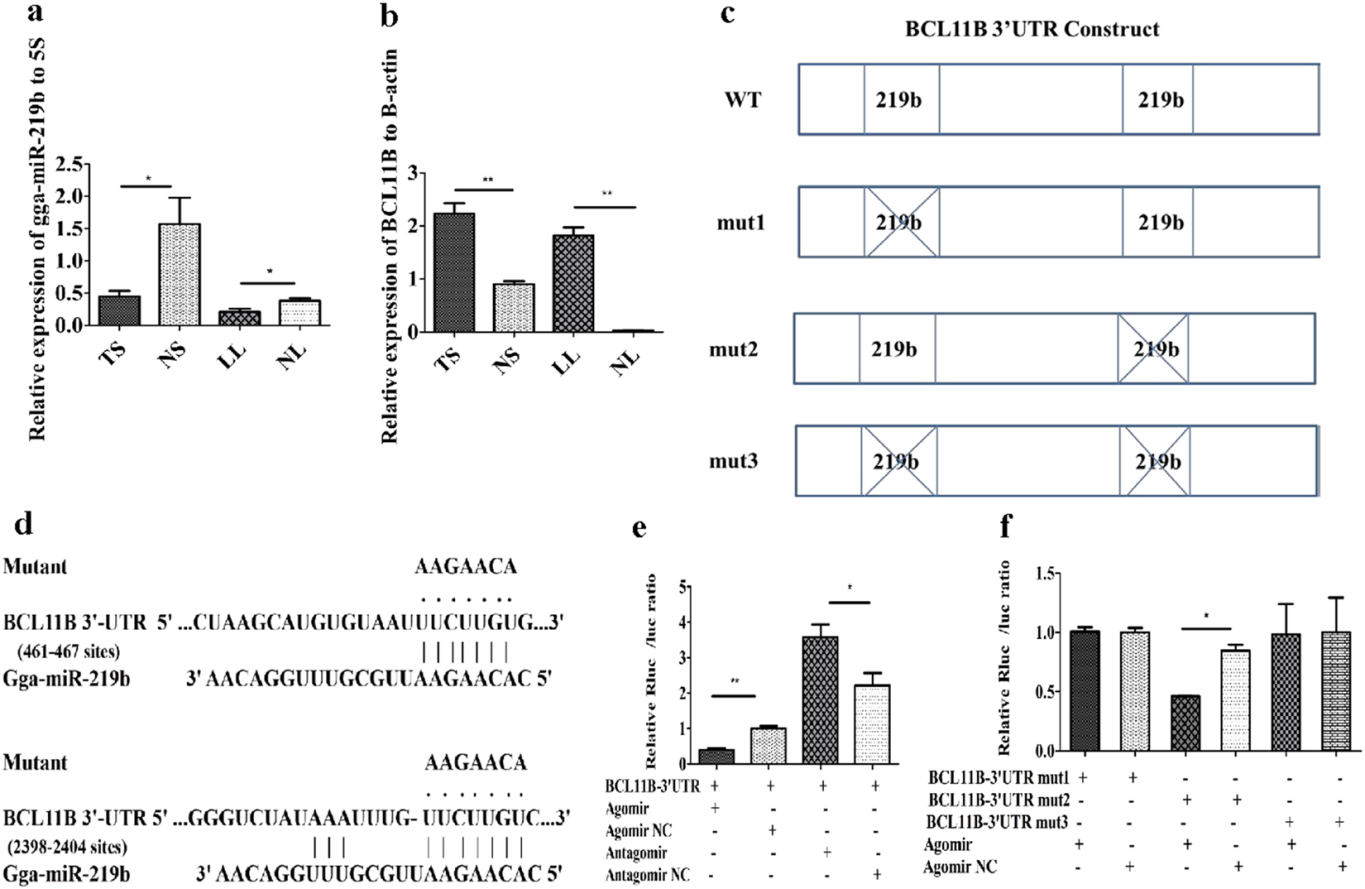
Validation of the predicted gga-miR-219b target gene. (**a,b**) Expression level of gga-miR-219b (**a**) and BCL11B (**b**) in non-tumorous spleen (NS), tumorous spleen (TS), non-tumorous liver (NL) and MD lymphoma from liver (LL) (n = 8). (**c**) Schematic diagram of gga-miR-219b candidate binding sites with the BCL11B 3′-UTR as well as mutants interrupting the binding sequences predicted by miRDB and TargetScan. (**d**) Nucleotide sequences of the wild-type and mutated gga-miR-219b binding sites located in the 3′-UTR of BCL11B. (**e,f**) Luciferase reporter assays in HEK293T cells transfected with reporter vectors containing either the wild-type (**e**) or mutated BCL11B-3′UTR (**f**) (n = 5). Differences between two groups were analysed by Student’s *t*-test with the SAS system. The data are expressed as the mean ± S.E. *P < 0.05. **P < 0.01.

BCL11B was predicted to be a target of gga-miR-219b by searching target genes in miRDB and TargetScan. The differential expression of gga-miR-219b and BCL11B was detected between tumorous tissue and non-infected controls by qRT-PCR. Gga-miR-219b expression was downregulated in tumorous spleen and liver compared with that in non-tumorous samples. In contrast, BCL11B expression was upregulated in tumorous spleen and liver compared with non-tumorous samples (Fig. 2a,b).

BCL11B has two putative binding sites of gga-miR-219b within its 3′-UTR. A dual-luciferase reporter assay was performed to verify whether BCL11B was a direct target gene of gga-miR-219b using the HEK293T cell line. Wild-type and mutant BCL11B-3′ UTR-containing putative binding sites were separately cloned into the pmiR-reporter vector downstream from the luciferase gene (Fig. 2c,d). Three mutant vectors were constructed to verify the two putative binding sites of miR-219b. The first one (BCL11B-3′UTR mut1) was only mutated at the 461-467 sites; the second one (BCL11B-3′UTR mut2) was only mutated at the 2398-2404 sites; the third one (BCL11B-3′UTR mut3) was mutated at both sites (Fig. 2c,d). We cotransfected HEK293T cells with the gga-miR-219b agomir or antagomir together with the reporter vector containing the wild-type or mutated 3′-UTR of BCL11B. The luciferase activity was significantly reduced by 61% when the gga-miR-219b agomir was cotransfected with the wild-type BCL11B 3′-UTR-containing vector. The luciferase activity was significantly decreased by 41% when the gga-miR-219b agomir was cotransfected with the BCL11B-3′UTR mut2 vector. In contrast, cotransfection of the agomir with the BCL11B-3′UTR mut1 or mut3 vector did not affect the luciferase activity (Fig. 2e,f). These results demonstrated a significant interaction of gga-miR-219b with BCL11B and that the 461–467 site in the seed region of BCL11B was the binding site for gga-miR-219b.

### Gga-miR-219b affected BCL11B expression at the transcriptional and translational level

**Figure 3:**
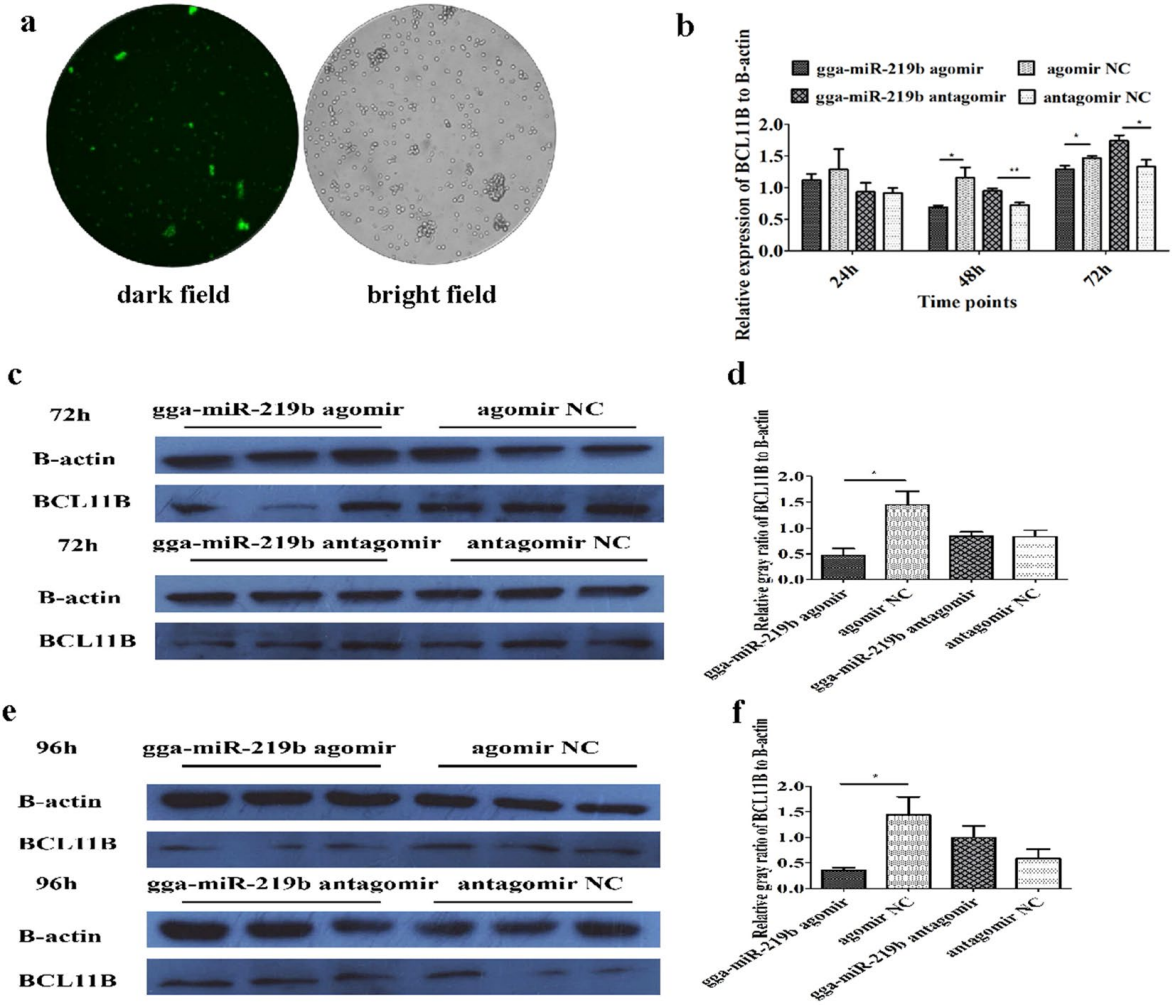
Effect of gga-miR-219b on BCL11B at the transcriptional and translational level. (**a**) Depiction of the miRNA agomir or antagomir transfection efficiency. To show transfection efficiency of the agomir or antagomir, a transfection reagent was used to transfect MSB1 cells with FAM-labelled agomir NC. A fluorescence microscope was used to observe the cells, which were illuminated under a bright field or dark field with magnification at 100×. (**b**) mRNA expression level of BCL11B after transfection with the miRNA agomir, antagomir or respective NC into MSB1 cells at 24 h, 48 h and 72 h (n = 4). (**c**–**f**) Protein expression level of BCL11B after transfection with the miRNA agomir, agomir NC, antagomir or antagomir NC into MSB1 cells at 72 h (**c**,**d**) and 96 h (**e**,**f**) (n = 3). The results of western blotting (**c,e**) and a diagram depicting the grey-scale values of BCL11B relative to β-actin are shown (**d,f**). The blots were cropped and full length blots were presented in Supplementary Figs S8–S13. Differences between two groups were analysed by Student’s *t*-test with the SAS system. The data are expressed as the mean ± S.E. *P < 0.05. **P < 0.01.

To confirm that BCL11B was the target gene of miR-219b, we detected the expression of BCL11B in MSB1 at the transcriptional and translational level after gga-miR-219b agomir and antagomir transfection. The transfection efficiency reached up to 70% (Fig. 3a). We examined BCL11B expression at 24 h, 48 h and 72 h post transfection. The mRNA level of BCL11B was remarkably downregulated at 48 h and 72 h post-agomir transfection and upregulated at 48 h and 72 h post-antagomir transfection (Fig. 3b). The protein expression of BCL11B was significantly lower in the agomir transfection group than in the agomir NC transfection group at 72 h and 96 h. Moreover, its expression displayed an upward trend in the antagomir transfection group at 96 h post transfection (Fig. 3c–f).

### BCL11B knockdown reduced MSB1 cell proliferation by promoting apoptosis, not by cell cycle arrest

**Figure 4:**
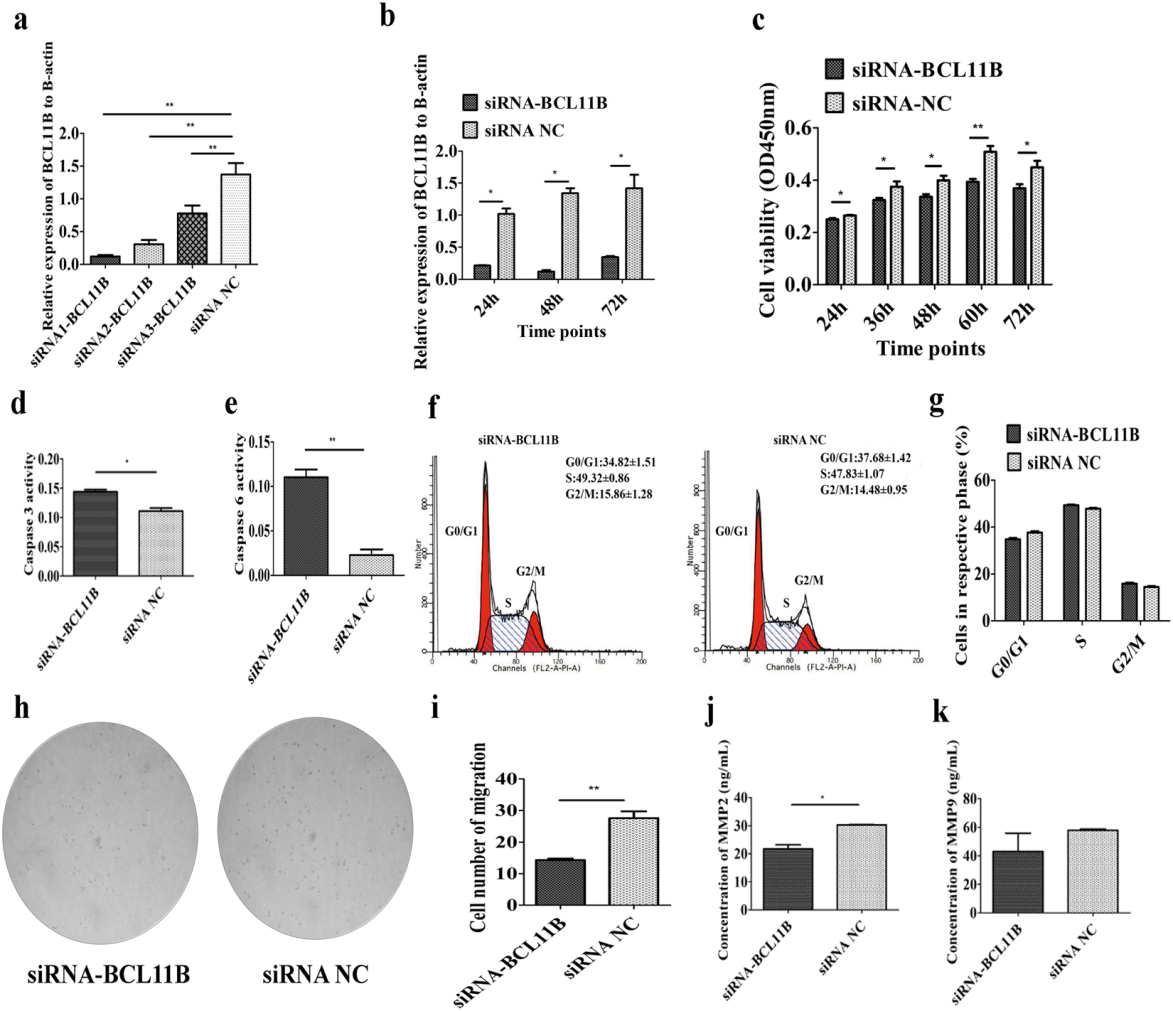
Effect of BCL11B knockdown on cell proliferation, migration and invasion in MSB1 cells. (**a**) Interference efficiency of three siRNAs designed to interfere with BCL11B determined by qRT-PCR (n = 4). (**b**) Diagrams of the siRNA-BCL11B interference efficiency on BCL11B determined by qRT-PCR (n = 4). (**c**) Effect of BCL11B knockdown on MSB1 cell proliferation. Cell proliferation was detected by CCK-8 assay at 24 h, 36 h, 48 h, 60 h and 72 h after transfection with siRNA-BCL11B and siRNA NC (n = 5). (**d,e**) Effect of BCL11B knockdown on MSB1 cell apoptosis. The activity of caspase-3 (**d**) and caspase-6 (**e**) was detected after transfection with siRNA-BCL11B and siRNA NC (n = 3). (**f**) Representative histograms depicting cell cycle profiles of MSB1 cells transiently transfected with siRNA-BCL11B and siRNA NC (n = 3). (**g**) Proportion of cells in various phases of the cell cycle (n = 3). (**h**) Representative images depicting cell migration profiles of MSB1 cells transiently transfected with siRNA-BCL11B and siRNA NC (n = 2). (**i**) Effect of BCL11B knockdown on MSB1 cell migration. Transwell migration assay of MSB1 cells was performed after transduction of siRNA-BCL11B and siRNA NC (n = 2). (**j,k**) Protein level of MMP2 (**j)** and MMP9 (**k)** after transduction of siRNA-BCL11B and siRNA NC (n = 4). Differences between two groups were analysed by Student’s *t*-test with the SAS system. The data are expressed as the mean ± S.E. *P < 0.05. **P < 0.01.

We designed three siRNAs to interfere with BCL11B expression and chose siRNA1-BCL11B, whose interference efficiency was the highest (hereafter called siRNA-BCL11B) (Fig. 4a). Its interference efficiency was 79% at 24 h, 91% at 48 h and 76% at 72 h post-transfection (Fig. 4b). Cell proliferation was significantly inhibited at 24 h, 36 h, 48 h, 60 h and 72 h post-siRNA transfection (Fig. 4c). Cell apoptosis showed an increasing trend at 48 h post-transfection in MSB1 cells with BCL11B knockdown (Supplementary Fig. S3). The activity of downstream effectors caspase-3 and caspase-6 was significantly increased at 48 h post-siRNA-BCL11B transfection (Fig. 4d,e). However, there was no significant change in the cell cycle after BCL11B interruption (Fig. 4f,g). To rule out the possibility of off-target effect, we also chose another siRNA (siRNA3-BCL11B) to inspect its effect on cell function. The results indicate that this siRNA had no effect on cell proliferation, apoptosis and cycle (Supplementary Fig. S16).

### BCL11B knockdown reduced MSB1 cell migration and invasion

We simultaneously detected migration and expression of the MMP2 and MMP9 genes after BCL11B knockdown in MSB1 cells. The migration cell number was markedly lower when siRNA-BCL11B was introduced (Fig. 4h,i). siRNA3-BCL11B had no effect on cell migration (Supplementary Fig. S16). In addition, mRNA expression of MMP2 and MMP9 was remarkably downregulated in the siRNA-BCL11B transfection group (Supplementary Fig. S4). The protein levels of both MMP2 and MMP9 were decreased after BCL11B knockdown at 48 h (Fig. 4j,k, Supplementary Fig. S15). mRNA expression of MMP2 and MMP9 had no obvious change post transfection of siRNA3-BCL11B (Supplementary Fig. S16).

### Gga-miR-219b mediated MSB1 cell apoptosis through influencing gene expression levels in the mitochondrial and death receptor pathways

**Figure 5:**
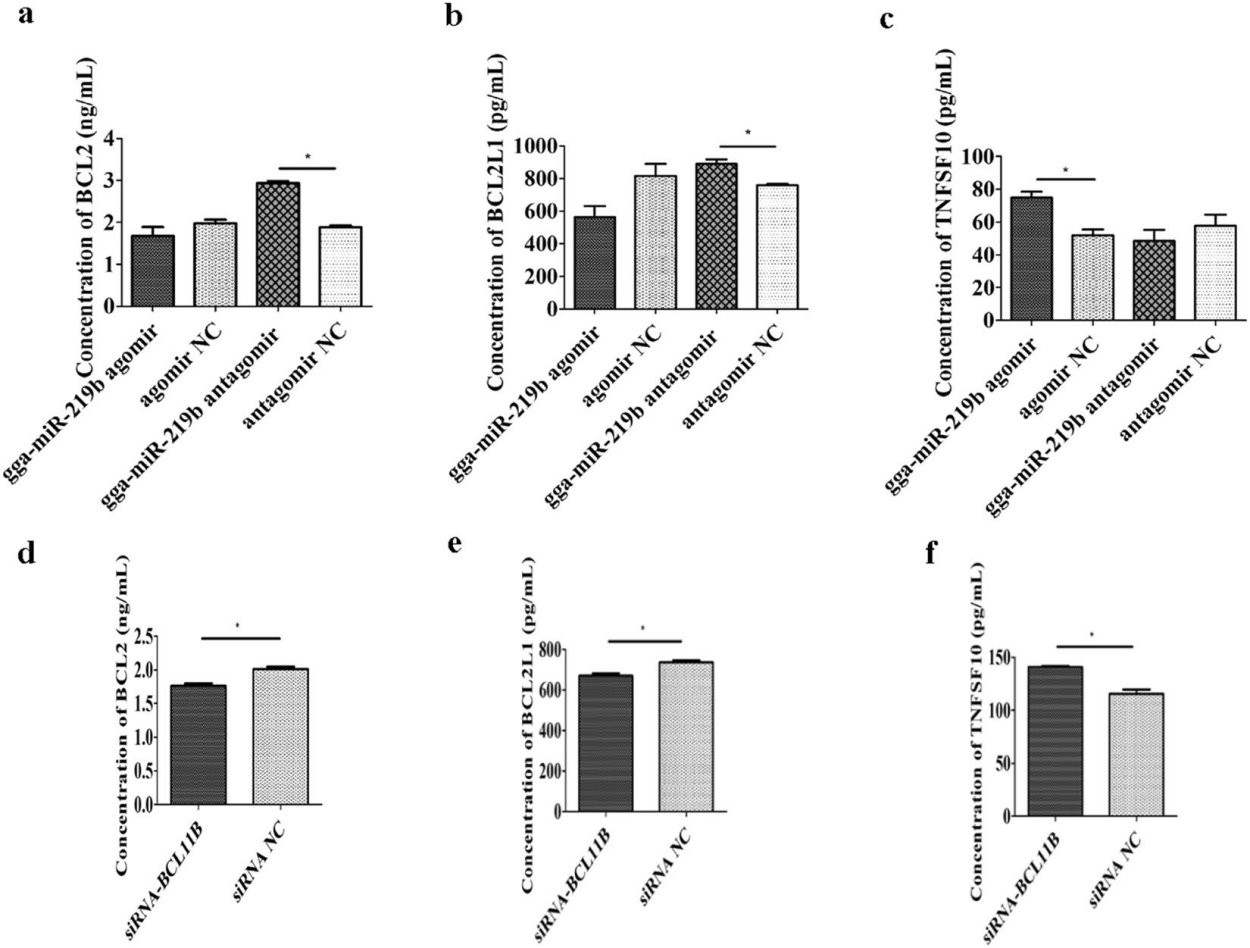
Effect of gga-miR-219b and BCL11B on gene expression at the protein level in the mitochondrial and death receptor pathways. (**a–c**) Protein level of BCL2 (**a**), BCL2L1 (**b**) and TNFSF10 (**c**) in the apoptosis pathways by ELISA after transient transfection with the gga-miR-219b agomir, antagomir or respective NC at 48 h (n = 3). (**d–f**) Protein level of BCL2 (**d**), BCL2L1 (**e**) and TNFSF10 (**f**) in the apoptosis pathways by ELISA after transient transfection with siRNA-BCL11B and siRNA NC at 48 h (n = 3). Differences between two groups were analysed by Student’s *t*-test with the SAS system. The data are expressed as the mean ± S.E. *P < 0.05. **P < 0.01.

The results above showed that both gga-miR-219b overexpression and BCL11B knockdown induced tumor cell apoptosis. To further elucidate the mechanism of apoptosis mediated by gga-miR-219b, we detected the expression of genes involved in apoptosis pathways, including the intrinsic mitochondrial pathway and death receptor pathway, at the transcriptional and translational level. First, at the transcriptional level, the expression of BCL2 was remarkably decreased at 48 h in the post-agomir transfection group, while it was significantly increased at 72 h in the post-antagomir transfection group; BCL2L1 expression was significantly decreased at 24 h and 48 h post-gga-miR-219b agomir transfection. In addition, TNFSF10 expression was markedly increased in the agomir transfection group at 72 h, while it was obviously decreased in the antagomir transfection group at 24 h and 72 h (Supplementary Fig. S5). Correspondingly, the expression of these genes was also detected when BCL11B expression was interrupted. The expression levels of BCL2 and BCL2L1 showed a decreasing trend, while TNFSF10 was upregulated slightly but significantly in the siRNA-BCL11B transfection group (Supplementary Fig. S6). Moreover, at the translational level, BCL2 and BCL2L1 protein levels in cell lysates were downregulated in the agomir transfection group and significantly upregulated in the antagomir transfection group; TNFSF10 was remarkably increased in the agomir transfection group at 48 h (Fig. 5a–c). After BCL11B knockdown, BCL2 and BCL2L1 protein levels were decreased slightly but significantly; additionally, TNFSF10 was significantly increased at 48 h post transfection (Fig. 5d–f). In combination, these results suggested that gga-miR-219b mediated tumor cell apoptosis by influencing gene expression in the apoptosis pathways.

### Gga-miR-219b and BCL11B affected Meq expression

**Figure 6:**
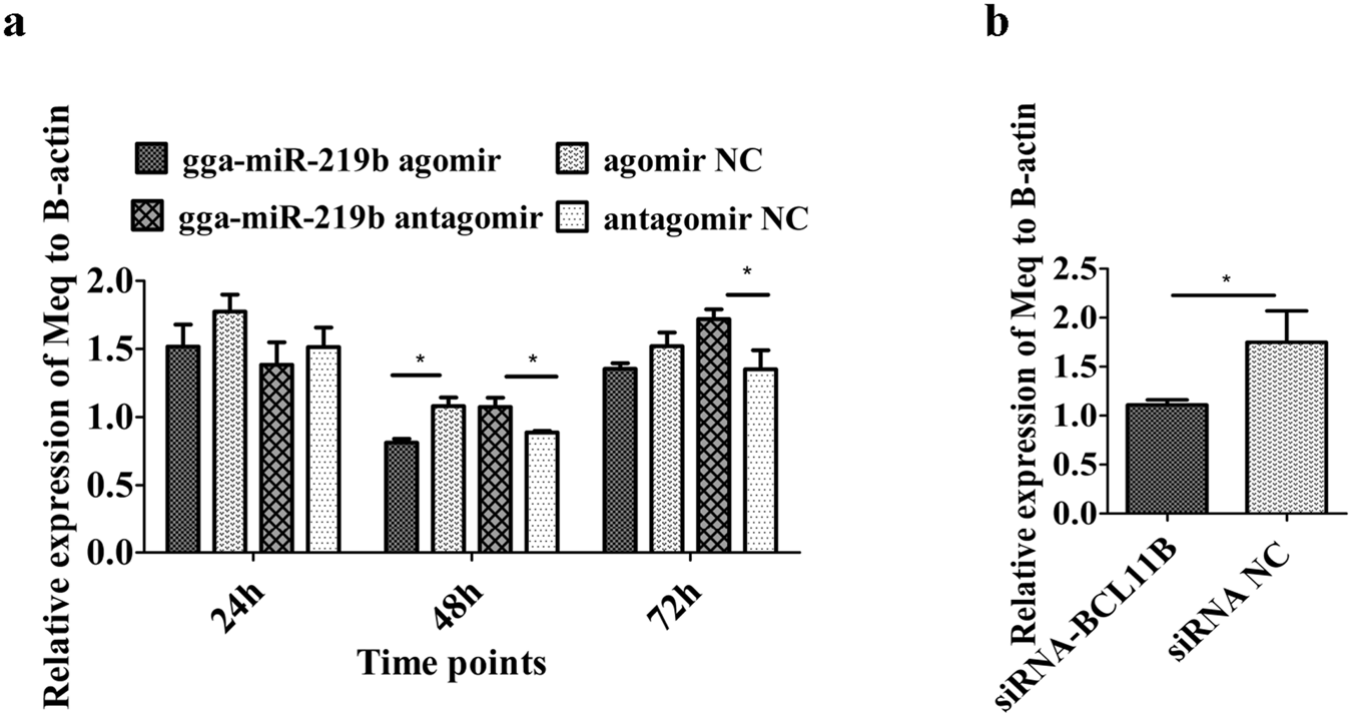
Effect of Meq gene expression on MD lymphoma transformation. (**a**) mRNA expression level of Meq after transfecting the miRNA agomir, antagomir or respective NC into MSB1 cells at 24 h, 48 h and 72 h (n = 4). (**b**) mRNA expression level of Meq after transfecting siRNA-BCL11B and siRNA NC into MSB1 cells at 48 h (n = 4). Differences between two groups were analysed by Student’s *t*-test with the SAS system. The data are expressed as the mean ± S.E. *P < 0.05. **P < 0.01.

Meq is an essential oncogene of MDV and is critical for viral pathogenicity. We found that expression of Meq could be affected by gga-miR-219b and BCL11B. When the gga-miR-219b agomir was introduced into MSB1 cells, Meq was greatly decreased at 48 h, while it was increased at 48 h and 72 h after antagomir transduction (Fig. 6a). When BCL11B was knocked down, Meq expression was remarkably reduced at 48 h (Fig. 6b).

## Discussion

Increasing evidence suggests that miRNAs play a critical role in tumorigenesis^21, 22^. The deregulation of miRNAs in the cellular pathway, acting as negative regulators of oncogenes or tumor suppressors, results in carcinogenesis and cancer progression^23^ and promotes tumor development^4, 24, 25^. It is reported that human hsa-miR-219 was involved in oligodendrocyte and neural precursor differentiation as well as carcinogenesis in acute myeloid leukaemia, pancreatic cancer, breast cancer, hepatocellular carcinoma (HCC) and glioblastoma^6, 26–30^, functioning as a tumor suppressor. The hypermethylation of hsa-miR-219 is related to early-stage tumor development in HCC patients. The methylation-dependent downregulation of hsa-miR-219 was found in HCC cell lines compared with that in normal liver tissue^31^. Overexpression of hsa-miR-219 in MCF-7 breast cancer cells results in accentuated expression of apoptosis- and proliferation-related anti-viral immunomodulators of the Jak-STAT and NF-ƙβ pathways^30^. Additionally, hsa-miR-219-5p was found to be regulated by IL-3, GM-CSF and G-CSF in acute myeloid leukaemia^6^, and it inhibits HCC cell proliferation through cell cycle arrest by targeting glypican-3^28^. Furthermore, hsa-miR-219-1-3p also inhibited cell proliferation, which is associated with a decrease in cyclin D1 and Akt and Erk pathway activation in pancreatic cancer cell lines^26^.

In chickens, gga-miR-219b was first identified in lung and trachea infected with avian influenza virus^32^. To our knowledge, the research on gga-miR-219b is very limited. In the current study, we found that gga-miR-219b might inhibit cell proliferation by promoting apoptosis of MSB1 cells. B-cell chronic lymphocytic leukaemia/lymphoma 11B (BCL11B) belongs to the BCL family, which is composed of BCL11A and BCL11B. They both encode a Krüppel-like C2H2 zinc finger protein, act as transcriptional factors and are involved in immune system malignancies. BCL11B is involved in T cell lineage commitment and maintenance. BCL11B-deficient mice showed stage-block in double-negative CD4^−^CD8^−^ thymocytes, which suggested that BCL11B is a critical regulator of both differentiation and survival during thymocyte development^33^. Moreover, BCL11B was recently found to be required for group 2 innate lymphoid cells, which play important roles in innate immunity by producing type 2 effector cytokines^34^. As a transcriptional factor, BCL11B promotes activation of interleukin-2 (IL-2)^35, 36^.

BCL11B not only plays an important role in thymocyte development but is also implicated in lymphoproliferative diseases^37–39^. BCL11B monoallelic deletions or missense mutations occurred across each of the major molecular subtypes of T-ALL, which suggested that BCL11B is a haploinsufficient tumor suppressor in human thymocyte transformation^38^. Suppression of BCL11B by siRNA selectively induced apoptosis in transformed T cells, whereas normal mature T cells remained unaffected, which made BCL11B an attractive therapeutic target in T-cell malignancies^37^.

In this study, when BCL11B expression was suppressed by siRNA, proliferation of the tumorous cell line MSB1 was effectively inhibited. There are two major signalling pathways that induce apoptosis, including the intrinsic death pathway and extrinsic death pathway^40–42^. The intrinsic death pathway, also termed the “mitochondrial” or “stress” pathway, is mainly regulated by the BCL-2 family. This pathway is initiated by BH3-only proteins, which inactivate the BCL-2 like proteins such as anti-apoptotic genes BCL2 and BCL2L1, resulting in Bax inhibition. Bax induces cytochrome *c* release into the cytosol from damaged mitochondria, which could provoke activation of caspase-9 and subsequent effectors caspase-3, -6, and -7. The extrinsic death pathway is induced when a ligand of the tumor necrosis factor (TNF) family, such as TNFSF10 (TRAIL), binds to cognate death receptors. This pathway activates caspase-8 via adaptor proteins including FADD. Moreover, caspase-8 is sufficient to lead to apoptosis with subsequent effector caspases.

Some evidence has shown that BCL11B and BCL2L1 are commonly concurrent in several disease models. Expression levels of BCL11B and BCL2L1 have reported to be significantly upregulated in T-ALL patients, and BCL11B overexpression was speculated to play a role in anti-apoptosis in T-ALL cells through upregulating its downstream gene BCL2L1^39^. In the human T-ALL cell line Molt, when BCL11B was blocked by siRNA, BCL2L1 expression was found to be decreased, while TNFSF10 expression was increased^40, 43^. Our findings were in accordance with the known role of BCL2L1 in malignant transformation^37^. Another important apoptosis mediator, TNFSF10, was found to be involved in BCL11B deficiency-induced cell death. Its transcriptional and translational activation was found in MSB1 cells as a result of BCL11B inhibition. It was reported that BCL11B interacted with the metastasis-associated proteins MTA1, MTA2 and MTA3 within the NuRD complex, which indicated that BCL11B might specifically recruit the NuRD complex to the unknown targeted genes and repress gene expression^36, 44^. Moreover, this was confirmed by the finding that the BCL11B/NuRD complex was detected on the promoter of the p57KIP putative tumor-suppressor gene in neuroblastoma cells and the complex associated with the HIV-1 long terminal repeat^45, 46^. After BCL11B inhibition, TNFSF10 was activated at the transcriptional and translational level in tumor T-cell lines such as Jurkat and huT78^37^. From previous studies, it was speculated that TNFSF10 might be one of the BCL11B/NuRD target genes, at least in the T-cell lineage^37^. BCL2L1 and TNFSF10 are key genes in the mitochondrial pathway and death receptor pathway and both of them could be affected by BCL11B, and thus, we deduced that BCL11B could be involved in the two apoptosis pathways. Therefore, we speculated that BCL11B mediates apoptosis through affecting the expression level of genes in the mitochondrial pathway and death receptor pathway (Supplementary Fig. S7).

MiR-219 was reported to decrease migration in different tumor cell lines^26, 29^. Both gga-miR-219b agomir transfection and BCL11B interruption inhibited migration of MSB1 cells. Furthermore, the expression levels of MMP2 and MMP9, which are closely associated with tumor cell invasion^47^, were reduced under both circumstances. These results suggested that gga-miR-219b and BCL11B could affect migration and invasion.

Meq (MDV Eco Q fragment-encoded protein) is an important oncogene in the MDV genome that is consistently expressed in latent tumor cells^48^. Meq encodes a bZIP protein with a leucine zipper domain at the N-terminus and a proline-rich transactivation domain at the C-terminus. As a DNA-binding transcriptional factor, Meq could bind with cellular and viral genes by forming homodimers (Meq/Meq) and heterodimers (Meq/Jun) to regulate gene expression. It was found that the MDV lytic replication origin, promoter for Meq and promoter for ICP4 were enriched by Meq binding; in addition, Meq and c-Jun could be co-recruited to the chicken IL-2 promoter^49^. The Meq heterodimer usually binds with AP-1 like sites^49, 50^, while the homodimer binds with MERE-II like sites^50^. Meq/Meq homodimers affect transrepression or transactivation, and Meq/Jun heterodimers can transactivate target genes carrying AP-1 like binding sites. Moreover, homodimerization of Meq is required for T cell lymphoma induction^51^. Meq is one of the few viral genes expressed in lytic and latent infections and is highly expressed in both MD tumor tissue and T-lymphoblastoid cell lines derived from cultured lymphoma explants^51^.

**Figure 7:**
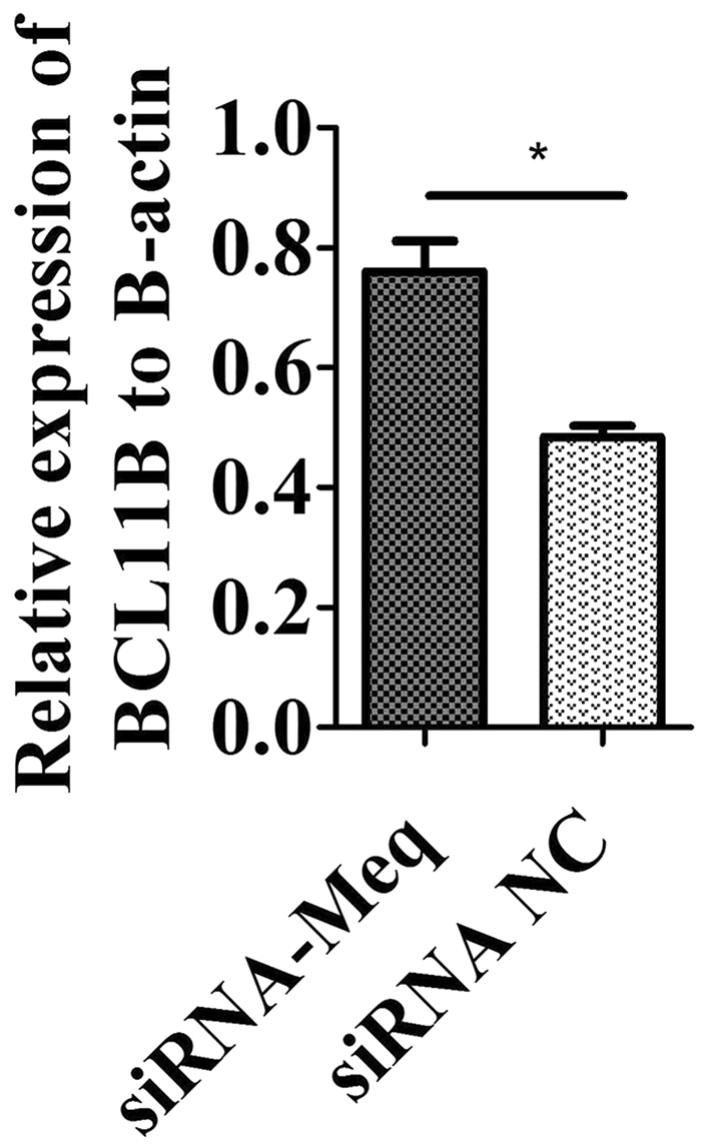
Expression level of BCL11B after Meq interference. mRNA expression level of BCL11B after transfecting siRNA-Meq or siRNA NC into MSB1 cells at 48 h (n = 3). The Meq targeting sequence is from Levy *et al*. (2005)^54^.

In our study, we found that both gga-miR-219b overexpression and BCL11B interruption reduced Meq expression. Both AP-1 like and MERE-II like motifs existed within 2 kb upstream of the BCL11B promoter. BCL11B expression was upregulated after Meq interference (Fig. 7), which indicates that there is a relationship between Meq and BCL11B. It was reported that the affinity of Meq towards Jun is much higher than that with itself ^52^, and perhaps this caused the upregulation of BCL11B after Meq knockdown. However, whether Meq binds to the BCL11B promotor to regulate its expression needs to be studied further.

In this study, we verified that BCL11B was the target gene of gga-miR-219b. Gga-miR-219b downregulated expression of BCL11B. Gga-miR-219b inhibited tumor cell proliferation, apoptosis, migration and invasion, acting as a tumor suppressor. The target of gga-miR-219b, BCL11B, promoted tumor cell proliferation, apoptosis, migration and invasion. Both gga-miR-219b and BCL11B mediated tumor cell apoptosis through influencing gene expression levels in the mitochondrial and death receptor pathways. Viral oncogene Meq expression might be mediated by both gga-miR-219b and BCL11B.

## Materials and Methods

### Ethics statements

All animal experiment procedures and sample collection strictly followed the protocols approved by the Animal Care and Use Committee of China Agricultural University (Approval ID: XXCB-20090209), and this study was carried out in strict accordance with the guidelines and regulations established by this committee.

### Sample collection

In previous study, 150 White Leghorn specific pathogen-free (SPF) chickens were used for experiment. One hundred randomly selected chickens were injected intraperitoneally with 2,000 plaque-forming units (PFU) of MDV GA strain and fifty chickens were infected with same dosage of diluent (0.2 mL) as controls at one day of age. The two groups were housed in separate isolators in different rooms. The trial period lasted until 56 days post-infection (d.p.i.). Eight MDV-infected tumorous spleens (whole spleens including tumor and adjacent non-tumor tissue), eight MD lymphomas from livers (dissected from adjacent non-tumor tissue), eight non-infected spleens, and eight non-infected livers were harvested during 31 to 55 days post-infection^20^. All tissues were stored in RNA fixer.

### Cell culture and miRNA transfection

The HEK293T cell line was grown in DMEM with 10% fetal bovine serum. MDV-transformed lymphoid cell line, MDCC-MSB1, kindly provided by Dr. C. Itakura, was cultured in RPMI-1640 with 10% fetal bovine serum. The two cell lines were maintained in a sterile incubator at 37 °C, 95% humidity and 5% CO2. FuGENE HD (Promega, Madison, WI) was used to transfect cell according to manufacturer’s instructions. The gga-miR-219b agomir, antagomir and corresponding NC were all purchased from GenePharma Company (GenePharma Co. Ltd., China).

### Searching for target genes of gga-miR-219b

Putative miRNA targets for gga-miR-219b were predicted by online software, Targetscan (http://www.targetscan.org/) and miRDB (http://mirdb.org/miRDB/). The targets predicted by two algorithms were taken into consideration. We combined the results from previous microarray data^53^ and referred to literatures to choose target genes for validation.

### Vector construction and luciferase reporter assays

To construct the luciferase reporter vectors, 3′-untranslated region (UTR) fragments (approximately 2700 bp) covering putative gga-miR-219b binding sites on the target mRNA of interest were amplified from genomic DNA, and restriction enzyme sites were added at primers (XhoI/NotI). The forward primer was 5′-CCGCTCGAGTTCCAAATTCACTAACAAAAAGGTACAT-3′. The downward primer was 5′-GAATGCGGCCGCCTCCCCCGCTAGGTTAAATTTC-3′. The amplified fragments were inserted into pmiR-RB-REPORT™ (RiboBio Co. Ltd.,China) within the XhoI/NotI sites.

Luciferase reporter experiments were performed in the HEK293T cell line. Cells were plated in a 96-well plate one day before transfection at 1.5 × 10^4^ cells/well and then pmiR-3′-UTR (100 ng) was cotransfected with gga-miR-219b agomir (100 nM), agomir NC (100 nM), antagomir (200 nM) or antagomir NC (200 nM). The relative luciferase activity was measured 48 h after transfection by Dual-Glo luciferase assay system (Promega) following manufacturer’s instructions.

To further verify the binding sites between gga-miR-219b and target mRNA, we also constructed mutated vectors. We constructed three mutated vectors concerning two seed regions in the BCL11B 3′-UTR. The first vector (BCL11B-3′UTR mut1) was only mutated at 461-467 sites; the second one (BCL11B-3′UTR mut2) was only mutated at 2398-2404 sites; the third one (BCL11B-3′UTR mut3) was mutated at both sites (Fig. 2c,d). The vectors with wild wild-type 3′-UTR (100 ng) or mutated 3′-UTR (100 ng) were cotransfected with gga-miR-219b agomir or agomir NC into HEK293T cells in a 96-well plate. The transfection and luciferase assay procedures were similar to those used above.

All constructs were verified by DNA sequencing. And all transfection experiments were performed in five replicates.

### Cell proliferation assay

The effect of gga-miR-219b on MSB1 cell proliferation was assessed using the Cell Counting Kit-8. The cells were seeded into 96-well plates (3 × 10^4^ cells/well) and transfected with gga-miR-219 agomir, antagomir or respective NC. Cell proliferation was detected at 24 h, 36 h, 48 h, 60 h and 72 h post-transfection. The absorbance at 450 nm was measured using a microplate spectrophotometer. All experiments were independently performed in five replicates.

### Cell apoptosis assay

For apoptosis assay, 5 × 10^5^ cells were seeded into six-well plates and transfected with gga-miR-219b agomir, antagomir or respective NC. Forty-eight hours after transfection, cells were harvested and stained with FITC Annexin V and Propidium Iodide (PI) (BD) for apoptosis assay using flow cytometry within one hour. Each assay was performed in triplicate.

### Caspase-3 and caspase-6 activity assay

The activity of caspase-3 and caspase-6 was determined using activity kit (Beyotime, China). Cell lysates were prepared after their respective treatment by incubating cells in extraction buffer for 30 min on ice. Lysates were centrifuged at 12,000 × *g* for 15 min, the supernatants were collected, and protein concentration was determined by Bradford protein assay kit. Cellular extracts were then incubated in a 96-well microtitre plate with 20 *ng* of Ac-DEVD-pNA (caspase-3 activity) or Ac-VEID-pNA (caspase-6 activity) for 2 h at 37 °C. Caspase activity was measured by cleavage of the Ac-DEVD-pNA or Ac-VEID-pNA substrate to pNA, the absorbance of which was measured at 405 nm. Caspase activity was calculated as concentration of product pNA/*ug*. All experiments were carried out in triplicate.

### Cell cycle assay

For cycle assay, 5 × 10^5^ cells were seeded into six-well plates and transfected with gga-miR-219b agomir, antagomir or respective NC. Forty-eight hours after transfection, cells were harvested and fixed with 70% ethanol and stored at 4 °C for twenty minutes. Fixed cells were washed with PBS, treated with RNase A (20 *μ*g/mL), and stained with PI (50 *μ*g/mL) for thirty minutes in the dark. The stained cells were analysed by flow cytometry. The cell debris and fixation artefacts were gated out, and the cell populations that were at the G0/G1, S and G2/M phases were quantified using the Modfit software. Each assay was performed in triplicate.

### Cell migration assay

For migration assay, 1.5 × 10^5^ cells were seeded into 24-well plates and transfected with gga-miR-219b agomir, antagomir or respective NC. After 48 h post-transfection, cells were collected and washed with PBS. 5 × 10^4^ cells in serum-free media were placed into the upper chamber of an insert (8-mm pore size; BD Bioscience, USA). Medium containing 20% fetal bovine serum was added to the lower chamber. After 16-18 hours of incubation, upper membrane was removed, and the cells that had migrated through the membrane were counted by microscope. Each assay was performed in two replicates.

### RNA interference

Twenty-one nucleotides of annealed, double-stranded siRNAs with d(TT) in the 3′-overhangs were synthesized by GenePharma. The targeting sequence of chicken BCL11B is confidential data that will be submitted for patent application. The siRNA NC was 5′-TTCTCCGAACGTGTCACGT-3′. Approximately 4 × 10^5^ cells were seeded into six-well plates and transfected with siRNA and siRNA NC (100 nM). Forty-eight hours after transfection, cells were harvested and stored at −80 °C for RNA isolation. Specific silencing efficiency was confirmed by qRT-PCR through four independent experiments.

### RNA isolation and real-time PCR analysis

Total RNA was isolated from frozen samples or MSB1 cells using TRIzol reagent according to manufacturer’s protocol. Total RNA was reverse transcribed by cDNA synthesis kit (miRACLE, USA) for miRNA expression detection. The qRT-PCR for miRNA was conducted by qPCR miRNA kit (miRACLE) according to manufacturer’s protocol. Specific forward primer for gga-miR-219b was 5′-CACAAGAATTGCGTTTGGACAA-3′. Specific forward primer for internal control 5 S was 5′-ACCGGGTGCTGTAGGCTTAA-3′. Each individual sample for gga-miR-219b detection was run in triplicate. The optimum thermal cycling parameters were 95 °C for 10 min, 40 cycles of 95 °C for 10 s, 57 °C for 20 s, and 72 °C for 1 min.

Total RNA was reverse transcribed by EasyScript First-Strand cDNA Synthesis SuperMix (TransGen, China) for specific gene detection. Real-time PCR reactions were performed by Power SYBR Green PCR Master Mix with the ABI 7500 system. The primers used for qPCR are listed in Table 1.

**Table 1:** Primers for genes used for qPCR.

Gene	Direction	Sequence
ß-actin	Forward	5′-GAGAAATTGTGCGTGACATCA-3′
	Reverse	5′-CCTGAACCTCTCATTGCCA-3′
	Forward	5′-CGTCGCAAGCAGGGAAAC-3′
BCL11B	Reverse	5′-CCCAGCGGGAAGTTCATC-3′
	Forward	5′-AAGTCACGACATCCCCAACAGC-3′
Meq^a^	Reverse	5′-TACATAGTCCGTCTGCTTCCTGCG-3′
	Forward	5′-TGAAACAGGAGATTTGGAT-3′
MMP2	Reverse	5′-CATTTTGGCTTTCTTGGA-3′
	Forward	5′-ACCTGGACCGTGCCGTGAT-3′
MMP9	Reverse	5′-TGCCTCGCCGCTGTAAAT-3′
	Forward	5′-CAGGAGCTGCTAAGTGTGCT-3′
BCL2L1^b^	Reverse	5′-CCCGGTTACTGCTGGACATT-3′
	Forward	5′-TTCCGTGATGGGGTCAACTG-3′
BCL2^b^	Reverse	5′-GTGGCAATGTTGTCCACCAG-3′
	Forward	5′-TGGCCGTCACCTACATCTAC-3′
TNFSF10^c^	Reverse	5′-TCAGCCACTCTGTCTTTGCT-3′

^a^Primer is from Heidari *et al*. (2008)^55^; ^b^Primer is from Subramaniam *et al*. (2013)^56^; ^c^Primer is from Li *et al*. (2008)^57^.

The relative expression level of genes was calculated with reference to expression of 5 S and β-actin. The results are described as fold change determined by the 2^−ΔΔCt^ method. The data are expressed as the mean ± standard error (SE).

### ELISA

Cell culture supernatants were collected and centrifuged at 1000 × *g* for 15 minutes to remove debris. Cells were suspended in PBS and subjected to ultrasonication. Then, 100 *μ*L sample was added to the wells in a microtiter plate pre-coated with antibody, and 10 *μ*L balance solution was dispensed into 100 *μ*L specimens. Next, 50 *μ*L conjugate was added to each well and thoroughly mixed, and 50 *μ*L substrate A and 50 *μ*L substrate B were subsequently added to each well after washing the microtiter plate five times. Finally, 50 *μ*L stop solution was added to each well after incubating for 10–15 minutes at 20–25 °C. The absorbance was immediately measured at 450 nm using a microplate reader.

### Western blot

Proteins were isolated using RIPA Lysis Buffer with phenylmethanesulfonylfluoride (PMSF). The concentration of proteins was determined by the BAC assay. Proteins were separated by 10% SDS-PAGE gels, and then transferred to polyvinylidene difluoride (PVDF) membranes. Membranes were blocked for 1 h and incubated overnight with primary antibody. After washing, the membranes were incubated for 1 h with second antibody. Proteins were detected using BeyoECL Plus and exposed to X-ray film.

### Statistical analysis

The data are expressed as the mean ± SE. All analyses were conducted using Student’s *t*-test with the SAS system. Differences were considered significant when P < 0.05.

## Electronic supplementary material

Supplementary Information
