# Supplementary material for: Gga-miR-219b targeting BCL11B suppresses proliferation, migration and invasion of Marek’s disease tumor cell MSB1
Source: Sci Rep. 2017 Jun 26;7:4247. doi: 10.1038/s41598-017-04434-w (PMC5484716; doi:10.1038/s41598-017-04434-w)

Gga-miR-219b targeting BCL11B, suppresses proliferation, migration and invasion of Marek's disease tumor cell MSB1

Chunfang Zhao[1], Xin Li[2], Bo Han[1], Zhen You[1], Lujiang Qu[1], Changjun Liu[3], Jiuzhou Song[4], Ling Lian[1*] and Ning Yang[1]*

Figure S1 Effect of gga-miR-219b on cell apoptosis in MSB1 cells.

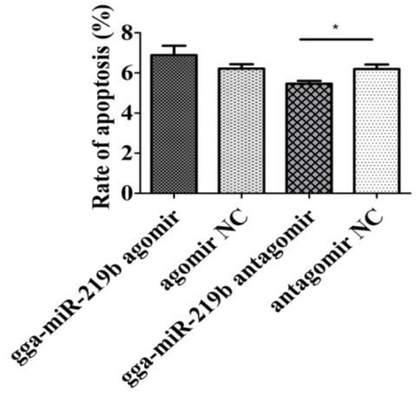

Figure S2 Relative mRNA expression of MMP2 and MMP9 after gga-miR-219b agomir or antagomir transfection at 24h, 48h and 72h.

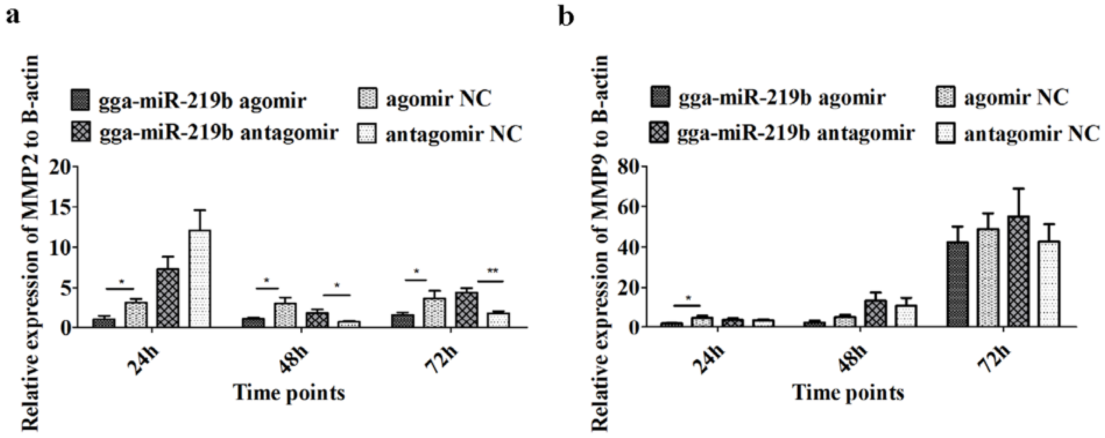

Figure S3 Effect of BCL11B knockdown on cell apoptosis in MSB1 cells.

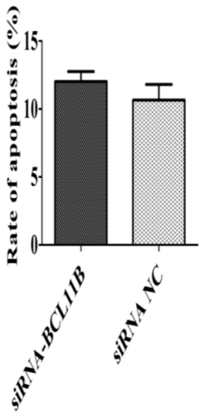

Figure S4 Relative mRNA expression of MMP2 and MMP9 after BCL11B knockdown at 48h.

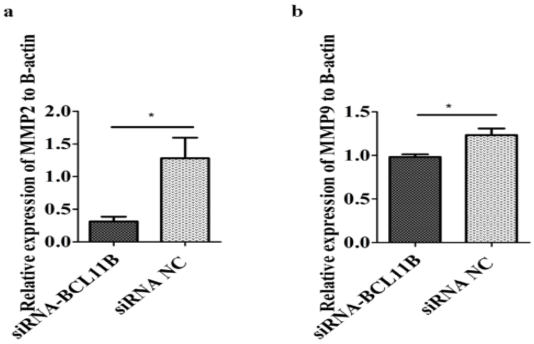

Figure S5 Relative mRNA expression of BCL2, BCL2L1 and TNFSF10 after gga-miR-219b agomir or antagomir transfection at 24h, 48h and 72h.

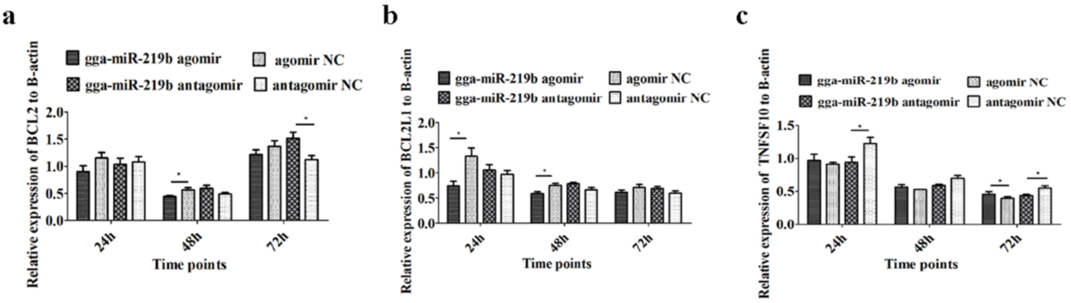

Figure S6 Relative mRNA expression of BCL2, BCL2L1 and TNFSF10 after BCL11B knockdown at 48h.

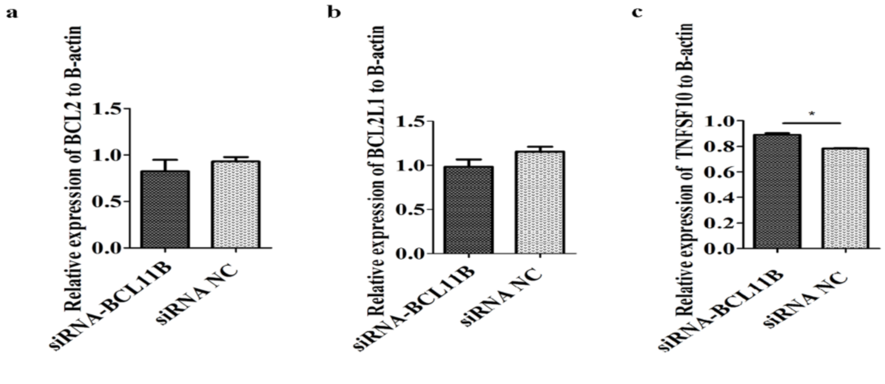

Figure S7 Speculated effect of BCL11B and gga-miR-219b on gene expression of apoptotic pathway in MSB1 cells [referred to reference 40 and 43].

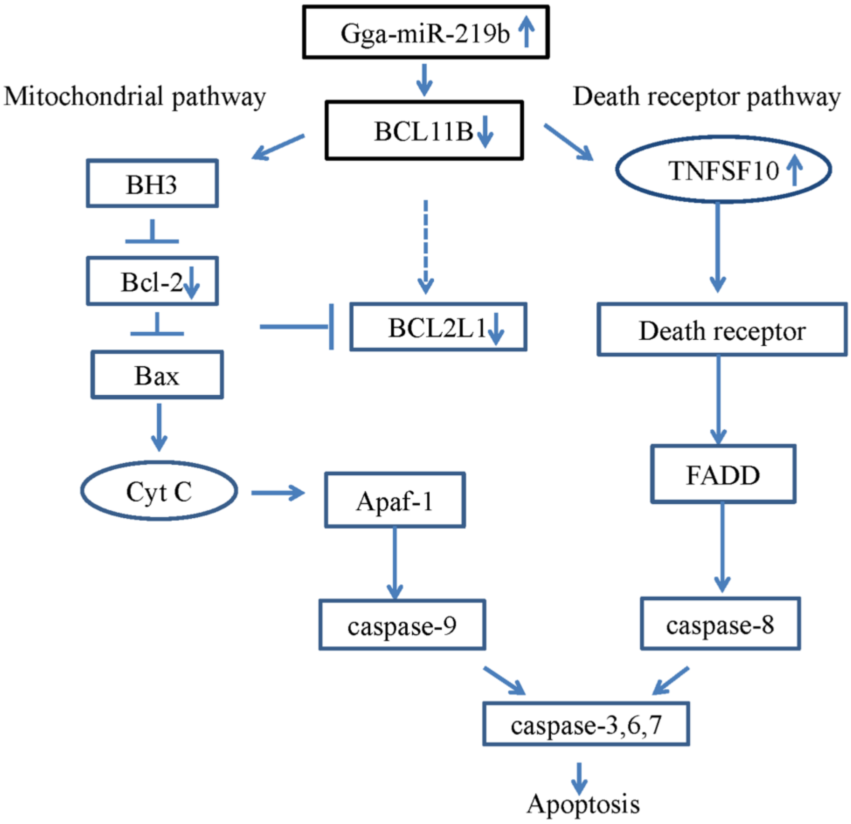

Figure S8 Western blotting of B-actin gene after gga-miR-219b agomir or agomir NC transfection at 72 h. It shows original western blotting bands of B-actin in Figure 3c.

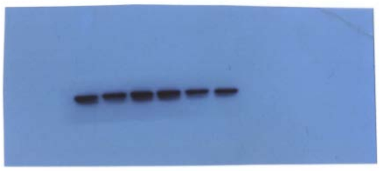

Figure S9 Western blotting of B-actin gene after gga-miR-219b antagomir or antagomir NC transfection at 72 h. It shows original western blotting bands of B-actin in Figure 3c.

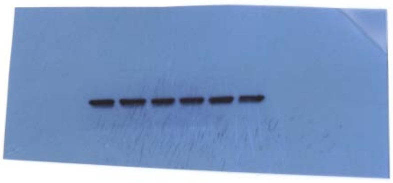

Figure S10 Western blotting of B-actin gene after gga-miR-219b agomir or

agomir NC transfection at 96 h. It shows original western blotting bands of B-actin in Figure 3e.

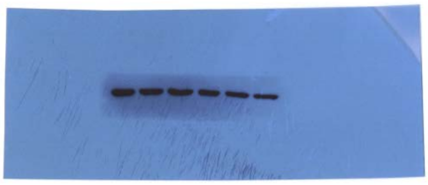

Figure S11 Western blotting of B-actin gene after gga-miR-219b antagomir or antagomir NC transfection at 96 h. It shows original western blotting bands of B-actin in Figure 3e.

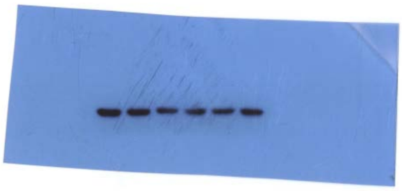

Figure S12 Western blotting of BCL11B gene after gga-miR-219b antagomir, antagomir NC, agomir or agomir NC transfection at 72 h. The upper image was the result of BCL11B gene after gga-miR-219b antagomir or antagomir NC transfection. The lower image was the result of BCL11B gene after gga-miR-219b agomir or agomir NC transfection. The antibody used for chicken was limited and polyclonal; the upper blot in the two images was the BCL11B gene according to protein marker. It shows original western blotting bands of BCL11B in Figure 3c.

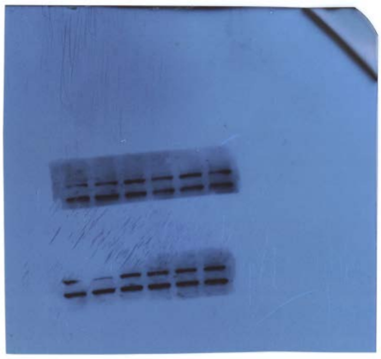

Figure S13 Western blotting of BCL11B gene after gga-miR-219b antagomir, anatagomir NC, agomir or agomir NC transfection at 96 h. The upper image was

the result of BCL11B gene after gga-miR-219b antagomir or antagomir NC transfection. The lower image was the result of BCL11B gene after gga-miR-219b agomir or agomir NC transfection. It shows original western blotting bands of BCL11B in Figure 3e.

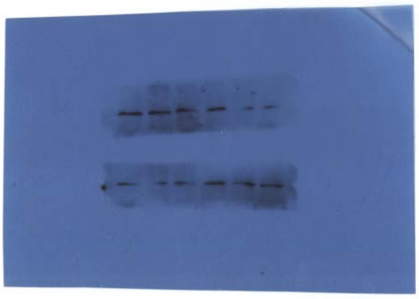

Figure S14 Protein expression level of MMP2 and MMP9 after transfection with the miRNA agomir, agomir NC, antagomir or antagomir NC into MSB1 cells at 48 h. (a) Western blotting of MMP2 and MMP9 gene. (b, c) The grey-scale values of MMP2 and MMP9 relative to ß-actin.

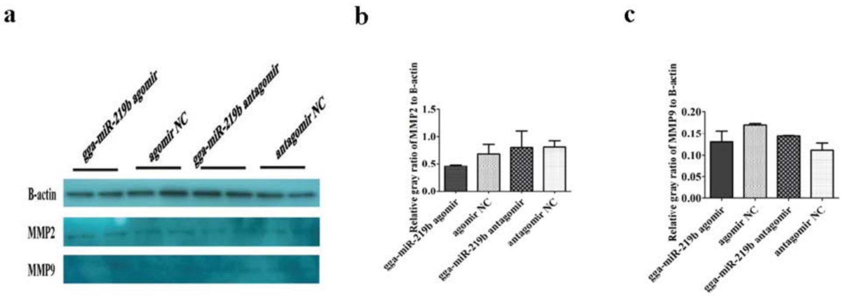

Figure S15 Protein expression level of MMP2 and MMP9 after transduction of siRNA-BCL11B and siRNA NC at 48h. (a) Western blotting of MMP2 and MMP9 gene. (b) The grey-scale values of MMP2 and MMP9 relative to ß-actin.

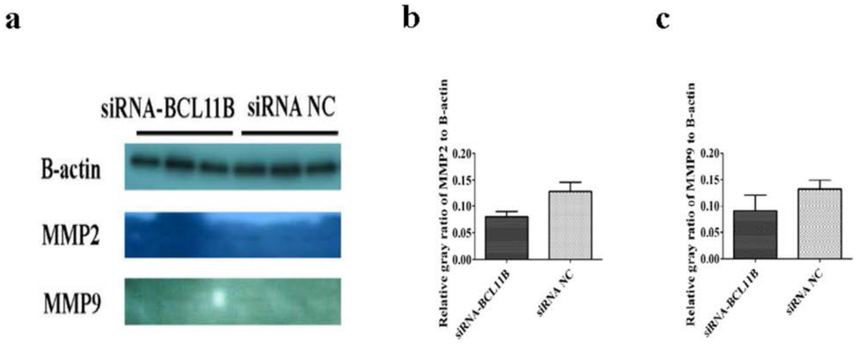

Figure S16 Effect of siRNA3-BCL11B on cell proliferation, apoptosis, cycle, migration and invasion in MSB1 cells. (a) Effect of siRNA3-BCL11B on MSB1

cell proliferation. Cell proliferation was detected by CCK-8 assay at 24 h, 36 h, 48 h, 60 h and 72 h after transfection with siRNA3-BCL11B and siRNA NC (n = 6). (b, c) Effect of siRNA3-BCL11B on MSB1 cell apoptosis. The activity of caspase-3 (b) and caspase-6 (c) was detected after transfection with siRNA3-BCL11B and siRNA NC (n = 3). (d) Representative histograms depicting cell cycle profiles of MSB1 cells transiently transfected with siRNA3-BCL11B and siRNA NC (n = 3). (e) Proportion of cells in various phases of the cell cycle (n = 3). (f) Representative images depicting cell migration profiles of MSB1 cells transiently transfected with siRNA3-BCL11B and siRNA NC (n = 2). (g) Effect of siRNA3-BCL11B on MSB1 cell migration. Transwell migration assay of MSB1 cells was performed after transduction of siRNA3-BCL11B and siRNA NC (n = 2). (h, i) Relative mRNA expression of MMP2 (h) and MMP9 (i) after transfection with siRNA3-BCL11B and siRNA NC at 48h (n = 3).

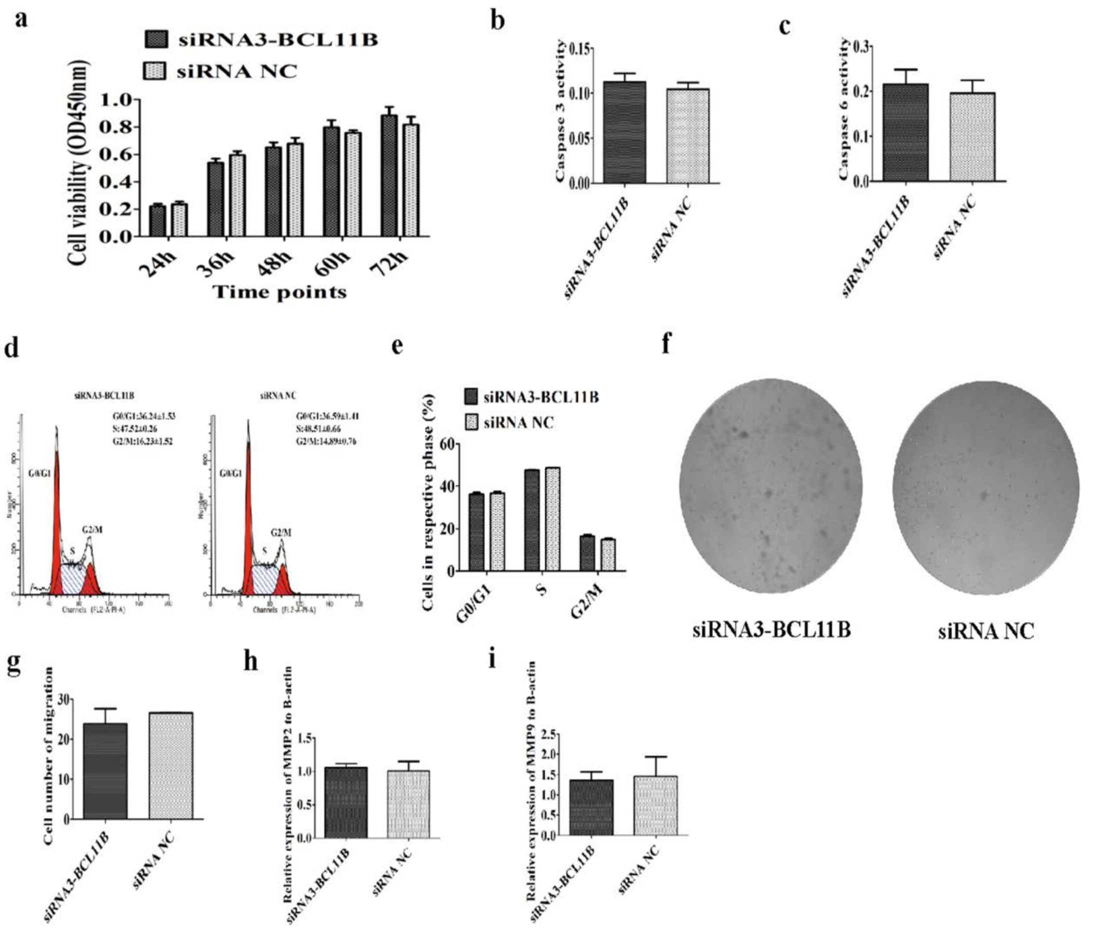

Supplement: Supplementary file 1 — Supplementary Information [file 41598_2017_4434_MOESM1_ESM.pdf]
